# Retrieval of HPV oncogenes E6 and E7 mRNA from cervical specimens using a manual open technology protocol

**DOI:** 10.1186/2193-1801-2-473

**Published:** 2013-09-18

**Authors:** Leonardo Martins Campbell, Denise Rocha Pitta, Angela Maria De Assis, Sophie Francoise Mauricette Derchain, Elisabete Aparecida Campos, Luis Otavio Zanatta Sarian

**Affiliations:** Gynecologic Oncology Department, Brasilia Mothers & Babies Hospital, SGAS 608 Mód A, Brasília, DF 70203-900 Brazil; Department of Obstetrics and Gynecology, Campinas State University, Campinas, Brazil

## Abstract

**Background:**

HPV oncogenes mRNA detection gains momentum as an adjuvant for HPV-related cervical abnormalities diagnosis, but is based on costly detection assays not allowing viral type targeting.

**Objective:**

To assess detection rate of HPV oncogenes E6/E7 mRNA from cervical specimens using a manual, open technology, fully customizable protocol and determine whether HPV-related epidemiological features influence mRNA retrieval. We reviewed literature and compared our retrieval rate with automated technologies.

**Methods:**

We used 60 samples positive for HPV DNA types 16, 18, 31 and/or 45. We extracted mRNA with a TRizol-based protocol, and tested mRNA purity and concentration using light absorbance. We reverse-transcribed mRNA into cDNA for E6/7 detection.

**Results:**

HPV oncogenes E6/E7 mRNA was retrieved from 36 (60%) out of 60 specimens. No HPV load-related clinical or epidemiological feature was significantly associated with mRNA retrieval. Presence of HPV-DNA 16/18 was associated with mRNA retrieval (OR = 9.08; 95% CI 1.26 to 65.32 for HPV 16; and 18.2; IC95% 1.86 to 391.44 for HPV 18).

**Conclusions:**

The open-technology protocol yielded an mRNA detection rate similar to that of automated technologies. Advantages are lower costs and target HPV type customization.

## Introduction

In the last two decades, the detection of Human papillomavirus (HPV) DNA in cervical samples has proven to be a good diagnostic and risk predictor tool for cervical intraepithelial neoplasia (CIN) and cervical cancer (Castle et al. 2011). Some studies suggest that HPV oncogenes E6 and E7 mRNA levels in the uterine cervix may be more specific early indicators of predisposition to carcinogenesis than DNA levels (Benevolo et al. 2011; Ratnam et al. 2011; Winer et al. 2009). This may be due to the fact that the oncogenic potential of the high-risk HPV types (hr-HPV) relies on the actions of oncoproteins coded by E6/E7, which bind to and modulate different gene products, in particular tumor suppressors p53 and pRb (Halfon et al. 2010; Kraus et al. 2006). These interactions can lead to cell cycle control disturbances, and defective DNA repair, causing genomic instability and increased risk of malignant transformation (Cattani et al. 2009; Münger & Howley 2002).

However, the potential use of HPV oncogenes' mRNA detection in clinical practice still needs robust confirmatory studies. One of the major limiting factors for the development of such studies is the fact that in order to determine mRNA levels in cervical specimens, one must rely on a handful of detection assays, which display different performance profiles. Most currently marketed assay options for mRNA detection use proprietary technology, and feature a pre-specified and non-customizable set of target HPV types. The PreTect HPV-Proofer (NorChip) and the NucliSENS Easy Q HPV (bioMérieux) are based on the same technology, and detect E6/E7 mRNA expression from the five most prevalent hrHPV types (16, 18, 31, 33 and 45). Other two tests aim at an expanded HPV types set: the TaqMan real-time PCR assay, which targets 12 high-risk (HPV16, 18, 31, 33, 35, 39, 45, 51, 52, 56, 58, 59) and two low-risk (HPV 6 and 11) types using E6/E7 region primers and probes in a duplex format (Andersson et al. 2011; Lindh et al. 2007), and the APTIMA HPV Assay (Gen-Probe) targets mRNA expression of the 14 most carcinogenic hr-HPV types (Burger et al. 2011; Dockter et al. 2009). However, the prevalence of HPV types may vary between female populations and the prespecified set of HPV types in a given test may prove inadequate in some epidemiological scenarios. Also importantly, the use of proprietary technology hinders the development of assay adaptations to laboratory and economic local conditions. The costs of the currently available commercial assays may not be compatible with resources available to many routine laboratories, especially in economically disadvantaged regions which, paradoxically, are heavily burdened by HPV-related diseases.

We intend to improve the efficacy and lower the cost of HPV oncogene detection, in this report we try to demonstrate how the detection rate of HPV oncogenes E6 and E7 mRNA from cervical specimens can be assessed using a manual open technology protocol, and determine whether the key epidemiological factors related to HPV viral load influence its mRNA retrieval rate.

## Materials and methods

### Sample and data collection

This study was carried out at the colposcopy clinics of Campinas State University (Unicamp), Brazil, a public health institution dedicated to comprehensive care for women, was approved by the local Ethics Committee (Protocol: N° CEP 723/2009) in compliance with the recommendations of the “Declaration of Helsinki” for guiding medical doctors in biomedical research involving human subjects. The samples consisted of cervical specimens, collected from women who underwent cervical diathermic conization due to HPV-induced squamous lesions at Unicamp’s cervical pathology clinics. Women were invited to participate while awaiting the conization procedure. Every research subject signed a written informed consent stating their compliance with the study, and an interview was performed concerning clinical and epidemiological aspects. After that, and immediately before the conization procedure, a cervical specimen was obtained with an endocervical brush, and stored in a 1-mL tube containing Specimen Transport Medium (STM, Qiagen Inc.).

In order to obtain the sample for these experiments we examined 117 specimens between April and December 2010. We first performed PCR reactions in order to ascertain the presence of HPV DNA using standard protocols (Swan et al. 1999). Of the 117 original cases 74 were positive for HPV DNA (PGMY 9/11). We next performed PCR reactions in order to ascertain the presence of DNA of the following HPV types: 16, 18, 31 and/or 45. Samples not harboring at least one of the HPV types of interest were discarded, and the patient excluded from the study. After completing 60 samples positive for at least one HPV type, we closed the accrual phase of the study (estimated sample size, considering 95% confidence intervals and 80% Beta power, for an estimated difference in mRNA levels of 20% between women harboring different HPV types, was 58). We then proceeded to the next phase of the study: detection of the mRNA in the samples.

#### mRNA extraction

As mentioned earlier, only samples positive for DNA of HPV types 16, 18, 31 and 45 were tested for mRNA. For mRNA extraction, an aliquot of 200 ul of STM was sampled (Qiagen) and centrifuged at 13,000 g for 10 min. The supernatant was removed and 1 mL of the TRizol™ reagent (Invitrogen, Carlsbad, USA) was added to the cellular pellet, and cells lysed by repeat pipetting and standing at room temperature for 5 min (TRizol is the most widely used reagent in the public setting in Brazil, hence its choice). Subsequently, 200 ul of chloroform was added and shaken vigorously, standing at room temperature for 3 min before centrifugation at 12,000 g for 15 min. The resultant aqueous layer was then transferred to a new tube. To the aqueous solution, a volume of 500 μl of isoamyl alcohol was added and mixed by vortex. The whole mixture stood at -80°C for 2 hours and 10 min at room temperature and centrifuged at 12,000 g for 10 min. Supernatant was then removed and 1 mL of iced ethanol was added and shaken vigorously. Finally, the mixture was centrifuged at 7,500 g for 5 min, the supernatant was removed and after the pellet of mRNA was dry, mRNA was eluted in 30 μl of mRNAase-free water (DEPC) and stored at -80°C pending analysis. The mRNA purity and concentration were determined by the absorbance at 260 nm (A260) and 280 nm (A280) using NanoDrop ND-1000 (Thermo Fisher Scientific, Waltham, MA).

#### Type-specific PCR for HPV 16, 18, 31 and 45 oncogenes E6 and E7 mRNA

PCR products were visualized using agarose gel electrophoresis. The reactions were performed separately according to the reagent mixture: five μl of DNA were added to a PCR mix containing 0.5 pmol of each primer (Table 1), specifically designed by the investigators for the present study, 4.0 mM MgCl2, 0.25 mM dNTPs and 5 units of Taq platinum in a final 50 μl volume . Every step of the PCR reactions was similar, except to annealing temperature which was different for each type HPV. During each PCR run, samples were tested altogether with one negative (water) and one positive control (obtained in prior studies). A 9-min denaturation step at 95°C was followed by 40 cycles of amplification. Each cycle included a denaturation step at 95°C for 1 min, a primer annealing step for each HPV type for 1 min, and a chain elongation step at 72°C for 1 minute. The final elongation step was prolonged by 7 min to ensure complete amplified DNA extension.

**Table 1:** **Primers designed for E6/E7 mRNA detection**

HPV	Region	Size	Sequence
16	E6	± 659 pb	5′ TAAAACTAAGGGCGTAACCG 3′
			5′ TCTATTTCATCCTCCTCCTCTG 3′
16	E7	± 491 pb	5′ ACTGTGTCCTGAAGAAAAGCAA 3′
			5′ AACCATCCATTACATCCCGT 3′
18	E6	± 608 pb	5′ TAGGTTGGGCAGCACATACT 3′
			5′ ATACTTGTGTTTCTCTGCGTCG 3′
	E7	± 358 pb	5′ CGACGCAGAGAAACACAAGTAT 3′
			5′ ATTGTTGCTTACTGCTGGGAT 3′
31	E6	± 606 pb	5′ AAGTAGGGAGTGACCGAAAGT 3′
			5′ ACAGTGGAGGTCAGTTGCC 3′
	E7	± 437 pb	5′ AGGCACGGCAAGAAAGACT 3′
			5′ AAAGAACCAGCCATTACACC 3′
45	E6	± 659 pb	5′ ATACTACATAAAAAAGGGTG 3′
			5′ TCGTAACACAACAGGTCAACA 3′
	E7	± 439 pb	5′ AGGCACGGCAAGAAAGACT 3′
			5′ AAAGAACCAGCCATTACACC 3′

#### CDNA transcription

The mRNA was reverse-transcribed into first strand cDNA (SuperScript III First Strand Synthesis System, Invitrogen) according to the manufacturer’s guidelines. The cDNA PCR product was amplified as described in the type-specific PCR section, although with a final volume of 25 μl and 2 μl of cDNA.

### Statistical analysis

All statistical analyses were performed with the R Environment for Statistical Computing (R Project). Significance was set at 95% (p = 0.05) and 95% confidence intervals were used (95% CI). We constructed a multivariate regression model assessing HPV oncogenes E6 and E7 mRNA retrieval according to clinical features and presence of HPV DNA of types 16, 18, 31 and/or 45. Odds ratios were obtained by exponentiation of the regression coefficients.

## Results

We retrieved oncogenes E6 and E7 mRNA from 36 (60%) of the 60 specimens previously found to be positive for the relevant HPV genotypes. None of the classically viral load - related clinical and epidemiological features that have been repeatedly reported in the medical literature was significantly associated with mRNA retrieval (Benevolo et al. 2011; Kraus et al. 2006). The presence of HPV 16 and 18 in the sample was positively associated with mRNA retrieval (OR = p < 9.08; 95% CI 1.26 to 65.32 for HPV 16; and 18.2; IC 95% 1.86 to 391.44 for HPV 18). Presence of other HPV genotypes was not associated to mRNA retrieval at the same rate, and associations were not significant at the 95% threshold (Table 2).

**Table 2:** **Recovery of viable E6/E7 mRNA according to key clinical features of the women and cyto-histological characteristics of the samples**

	mRNA E6 or E7						
Characteristic	neg	(%)	pos	(%)	OR	(95% CI)	p
Age							
<30	14	(58.3)	16	(44.4)	4.18	(0.87 to 20.05)	0.073
>30	10	(41.7)	20	(55.6)	1.0		
First intercourse							
<16	14	(58.3)	19	(52.8)	0.73	(0.2 to 2.72)	0.637
>16	10	(41.7)	17	(47.2)	1.0		
Lifetime sex partners							
>2	5	(20.8)	9	(25)	0.55	(0.11 to 2.69)	0.461
1-2	19	(79.2)	27	(75)	1.0		
Current smoker							
Yes	8	(33.3)	11	(30.6)	0.8	(0.2 to 3.18)	0.751
No	16	(66.7)	25	(69.4)	1.0		
Cytology							
ASC-H or HSIL	19	(79.2)	27	(75)	0.36	(0.06 to 2.02)	0.245
ASC-US or LSIL	5	(20.8)	9	(25)	1.0		
Histology							
CIN2-CIN3	21	(87.5)	35	(97.2)	5.43	(0.24 to 124.79)	0.290
Cervivitis-CIN1	3	(12.5)	1	(2.8)	1.0		
HPV Type-specific mRNA transcription* **							
DNA-HPV 16							
Positive	17	(40.4)	25	(59.6)	9.08	(1.26 to 65.32)	0.028
DNA HPV 18							
Positive	2	(28.5)	5	(71.5)	18.27	(1.86 to 392.44)	0.033
DNA HPV 31							
Positive	14	(54.4)	12	(45.6)	3.18	(0.68 to 14.89)	0.142
DNA HPV 45							
Positive	5	(50.0)	5	(50.0)	13.45	(0.73 to 246.64)	0.080

*As per study design samples negative for a given HPV type were not re-tested for that type-specific mRNA; **percentages in rows.

Table 3 lists key findings of major studies addressing mRNA detection rate in HPV DNA positive cervical specimens. Our detection rate of 60% is comparable to that reported by (Halfon et al. 2010) (68.5%) and (Ratnam et al. 2011) (68%), who used the automated NucliSens Easy Q HPV Assay BioMerieux and Aptima HPV detection assays, respectively. Those detection assays are targeted to HPV types 16, 18, 31, 33 and 45, which is basically the same set of HPV types addressed in our experiments, except for HPV 33. (Dockter et al. 2009) compared the performance of the fully automated Aptima HPV Assay DST with that of the semi-automated Aptima HPV Assay Tigris DST System. In samples previously tested for HPV DNA with Hybrid Capture 2, they achieved 92.6% and 91.9% E6/E7 mRNA detection rates, respectively. However, those assays target 14 HPV types. (Molden et al. 2005) and (Benevolo et al. 2011) using the Pre-Tec HPV Proofer NASBA reported 22.8% and 36.0% mRNA detection rates, respectively.

**Table 3:** **Studies assessing mRNA retrieval from HPV-DNA positive cervical samples using currently available detection assays**

Author	Year	Detection technique	Automation	HPV types included in assay	E6/E7 mRNA detection rate
Molden	2005	Pre-Tec HPV Proofer NASBA	Automated	16 18 31 33 45	98/429 (228%)
Catani	2009	NucliSens Easy Q HPV Assay BioMerieux	Automated	16 18 31 33 45	81/180 (45%)
Dockter*	2009	Aptima HPV Assay Tigris DST System	Semi-automated	16 18 31 33 35 39 45 51 52 56 58 59 66 and 68	407/443 (919%)
Dockter*	2009	Aptima HPV Assay DST	Automated	16 18 31 33 35 39 45 51 52 56 58 59 66 and 68	410/443 (926%)
Halfon	2010	NucliSens Easy Q HPV Assay BioMerieux	Automated	16 18 31 33 45	61/89 (68.5%)
Monsonego	2010	Aptima HPV Assay	Automated	16 18 31 33 35 39 45 51 52 56 58 59 66 and 68	456/4429 (10.3%)
Benevolo	2011	Pre-Tec HPV Proofer NASBA	Automated	16 18 31 33 45	162/464 (36%)
Ratnam	2011	Aptima HPV Assay	Automated	16 18 31 33 35 39 45 51 52 56 58 59 66 and 68	964/1418 (68%)
**Our study**	**2012**	**Homebrew low-cost TRizol protocol**	**Manual**	**16 18 31 45**	**36/60 (60%)**

*Both APTIMA tests were compared in the same publication.

## Discussion

In our study, using open technology protocols (which, thanks to their relatively low-cost are appropriate for low-resource settings), we retrieved viable oncogenes E6 and E7 mRNA in almost 60% of the HPV DNA positive cases. The extraction technique was completely manual. There are plenty of reports on this subject using automated our semi-automated detection techniques, with manufacturer prespecified sets of HPV types included in the assay. Our mRNA detection rate was comparable to those reported by most authors using automated mRNA detection techniques (Benevolo et al. 2011; Cattani et al. 2009). Using proprietary technology protocols, such as the PreTect HPV-Proofer (NorChip) and the NucliSENS Easy Q HPV (bioMérieux), studies similar to ours achieved overall mRNA retrieval rates ranging from 36% to 68% (Benevolo et al. 2011; Ratnam et al. 2011; Halfon et al. 2010; Cattani et al. 2009; Dockter et al. 2009; Molden et al. 2005; Monsonego et al. 2010). Therefore, our results seem to point out to a reliable and not too expensive way of detecting mRNA. Larger and prospective studies might well confirm the reliability of open technology protocols for mRNA detection and underscore its indications in clinical and laboratory practice. Also worth noting, open technology manual techniques could allow tailoring experiments to the HPV epidemiological profile (i.e. the most prevalent HPV types) of the population under scrutiny.

Our study has some limitations, including a lack of cytology – histology – mRNA detection which was not included at this time and this also limits the comparison of our retrieval technique with other methods. Therefore a comparison with pathological samples from other publications was also not possible at this time. Another limitation of our study is that we also did not perform a comparison with other assays using the same sample set. Finally, another caveat of this study was that we did not perform a breakdown of all HPV genotypes present in each samples (for times more than one), what could point out to an association of multiple genotypes and mRNA.

However, scientific evidence accumulated from virological, molecular, clinical and epidemiological studies has unequivocally demonstrated that cervical cancer is in fact a sequel from a long term unresolved infection by certain HPV genotypes (Castellsagué 2008). In the largest multinational studies performed so far, HPV types 16, 18, 31, 33 and 45 were shown to be the most prevalent types associated with cervical carcinomas, HPV 16 alone found in more than 50% of cases (Kraus et al. 2006; Bosch et al. 1995). In North India, HPV 16 was by far the commonest single type in all histological categories. Similar results have been reported in other studies (Bhatla et al. 2008). Studies using quantitative type-specific PCR for high-risk HPV 16, 18, 31, 33, and 45 and low-risk HPV 6 and 11 have shown that HPV 16 can reach much higher viral loads than the other types and that only for HPV 16 does increased viral load correlate with increased severity of cervical disease (Burd 2003). However, questions do remain as to how better oncogene mRNA transcription rates favor the carcinogenic potential of some HPV types, and to which individual patient characteristics may be involved in the DNA-to-mRNA transcription rates.

Despite the substantial resources spent in cytology screening and follow-up, cervical cancer is still the 10th most common cause of cancer death in European women. In Brazil by contrast, although rapidly declining, cervical cancer is one of the leading causes of mortality among women, and the cervical cancer burden is even heavier in less developed countries. Because cervical cancer is the only cancer that is almost completely preventable through regular screening and thus early treatment, improvement and expansion of existing screening strategies and technologies constitutes a main target of the European Council Recommendation on Cancer Screening (Boulet et al. 2008). However, many of the proposed screening strategies, due to resource constraints, may not be applicable worldwide. A test that can reliably differentiate between transient and persistent infections would allow additional effective cervical cancer policies targeting its prevention (Burger et al. 2011) which would be very welcomed. Detection of mRNA in cervical samples may probably serve this purpose, for the aforementioned reasons.

Current lines of evidence reinforce the biological reasons for detecting HPV oncogenes E6 and E7 mRNA. HPV screening cohort studies have shown that HPV DNA testing has a higher sensitivity than cytological testing for the detection of cervical lesions, although it has slightly lower specificity (Halfon et al. 2010). Its sensitivity for moderate dysplasia or worse is lower, but the specificity is higher (Cattani et al. 2009; Keegan et al. 2009; Szarewski et al. 2008). It is well-known that many cervical lesions with moderate or severe dysplasia will regress spontaneously. Only 31% of colposcopically visible lesions with severe dysplasia will progress to invasive cancer within 30 years (McCredie et al. 2008). Changes such as integration of viral DNA (which stabilizes the expression of E6 and E7) are virus specific, associated with the malignant progression of the tumor. Other changes include the alteration of cellular genes, leading to down-regulation of tumor suppressor genes and proapoptotic genes or upregulation of proto-oncogenes or antiapoptotic genes. These alterations reflect the effects of prolonged viral gene expression, in particular viral proteins E6 and E7 (Snijders et al. 2006). In productive HPV infections, which appear cytologically as LSIL and histologically as CIN1, the expression of viral E6 and E7 oncogenes is tightly regulated, with high-level expression only in suprabasal postmitotic cells. On the other hand, in high-grade CIN and cancer, E6 and E7 are expressed throughout the thickness of the cervical epithelium (Stanley 2008).

Given the fact that sustained viral oncogenes E6 and E7 expression is essential for the initiation and maintenance of the transformed phenotype induced by mucosal high-risk HPVs, several assays have been specifically developed for the detection of HPV E6⁄E7 mRNA (Snijders et al. 2010), despite the fact that positivity rates of the HPV mRNA test are one third that of HPV DNA tests. Increased knowledge concerning the molecular biology of cervical carcinogenesis raises expectations that biomarkers will result in a more accurate diagnosis of cervical cancer (Horvath et al. 2008). Moreover, the emergence of molecular medicine has resulted in the increased use of RNA in clinical diagnostics (Cuschieri et al. 2005). The identification of persistent infections has become a primary target for HPV mRNA testing because infections that do not regress are more likely to lead to cellular changes of the cervix, pre cancer lesions and/or invasive cervical cancer.

DNA and mRNA testing may be employed together for screening to take advantage of the combined higher sensitivity and specificity these tests have shown; patients would e.g. then be referred for a biopsy when both tests were positive (Halfon et al. 2010). Although the combined use of DNA and mRNA is a promising alternative for the improvement of molecular-biology based screening of cervical abnormalities, proprietary technology costs may preclude its implementation in low-resource settings. Furthermore, the use of assays targeted at a prespecified set of HPV types may be a waste of resources in regions with a different HPV type distribution. For the above mentioned reasons, the potential for clinical applicability of mRNA detection in low-resource settings seems to be large.

## Abbreviations

STM: Specimen transport medium
DEPC: Diethylpyrocarbonate water
PGMY: Primer system
ASC-H: Atypical squamous cells – cannot exclude HSIL
HSIL: High grade squamous intraepithelial lesion
ASC-US: Atypical Squamous Cells of Undetermined Significance
LSIL: Low grade squamous intraepithelial lesion
CIN2: Cervical intraepithelial neoplasia grade 2
CIN3: Cervical intraepithelial neoplasia grade 3
Cervicitis: Inflammation of the uterine cervix inflammation of the uterine cervix
CIN1: Cervical intraepithelial neoplasia grade 1.
